# FIGL1 and its novel partner FLIP form a conserved complex that regulates homologous recombination

**DOI:** 10.1371/journal.pgen.1007317

**Published:** 2018-04-02

**Authors:** Joiselle Blanche Fernandes, Marine Duhamel, Mathilde Seguéla-Arnaud, Nicole Froger, Chloé Girard, Sandrine Choinard, Victor Solier, Nancy De Winne, Geert De Jaeger, Kris Gevaert, Philippe Andrey, Mathilde Grelon, Raphael Guerois, Rajeev Kumar, Raphaël Mercier

**Affiliations:** 1 Institut Jean-Pierre Bourgin, UMR1318 INRA-AgroParisTech, Université Paris-Saclay, RD10,Versailles, France; 2 Université Paris-Sud, Université Paris-Saclay, Orsay, France; 3 Department of Plant Biotechnology and Bioinformatics, Ghent University, Ghent, Belgium; 4 VIB Center for Plant Systems Biology, Ghent, Belgium; 5 Department of Biochemistry, Ghent University, Ghent, Belgium; 6 VIB Center for Medical Biotechnology, Ghent, Belgium; 7 Institute for Integrative Biology of the Cell (I2BC), Commissariat à l’Energie Atomique et aux Energies Alternatives (CEA), Centre National de la Recherche Scientifique (CNRS), Université Paris-Sud, CEA-Saclay, Gif-sur-Yvette, France; National Cancer Institute, UNITED STATES

## Abstract

Homologous recombination is central to repair DNA double-strand breaks, either accidently arising in mitotic cells or in a programed manner at meiosis. Crossovers resulting from the repair of meiotic breaks are essential for proper chromosome segregation and increase genetic diversity of the progeny. However, mechanisms regulating crossover formation remain elusive. Here, we identified through genetic and protein-protein interaction screens FIDGETIN-LIKE-1 INTERACTING PROTEIN (FLIP) as a new partner of the previously characterized anti-crossover factor FIDGETIN-LIKE-1 (FIGL1) in *Arabidopsis thaliana*. We showed that FLIP limits meiotic crossover together with FIGL1. Further, FLIP and FIGL1 form a protein complex conserved from Arabidopsis to human. FIGL1 interacts with the recombinases RAD51 and DMC1, the enzymes that catalyze the DNA strand exchange step of homologous recombination. Arabidopsis *flip* mutants recapitulate the *figl1* phenotype, with enhanced meiotic recombination associated with change in counts of DMC1 and RAD51 foci. Our data thus suggests that FLIP and FIGL1 form a conserved complex that regulates the crucial step of strand invasion in homologous recombination.

## Author summary

Homologous recombination is a DNA repair mechanism that is essential to preserve the integrity of genetic information and thus to prevent cancer formation. Homologous recombination is also used during sexual reproduction to generate genetic diversity in the offspring by shuffling parental chromosomes. Here, we identified a novel protein complex (FLIP-FIGL1) that regulates homologous recombination and is conserved from plants to mammals. This suggests the existence of a novel mode of regulation at a central step of homologous recombination.

## Introduction

Homologous recombination (HR) is critical for the repair of DNA double-strand breaks (DSBs) in both mitotic and meiotic cells [1]. Defects in HR repair causes genomic instability, leading to cancer predisposition and various inherited diseases in humans [2]. During meiosis, HR promotes reciprocal exchange of genetic material between the homologous chromosomes by forming crossovers (COs). COs between the homologs constitute a physical link that is crucial for the accurate segregation of homologous chromosomes during meiosis [3]. COs also reshuffle parental genomes to enhance genetic diversity on which selection can act [4]. Failure or errors in HR at meiosis lead to sterility and aneuploidy, such as Down syndrome in humans [5,6].

During meiosis, HR is initiated by the formation of numerous programmed DSBs catalyzed by the topoisomerase-like protein SPO11 [7]. DSBs are resected to form 3’ single-stranded DNA (ssDNA) overhangs. A central step of HR is the search and invasion of an intact homologous template by the broken DNA end, which is catalyzed by two recombinases, RAD51 and its meiosis-specific paralog DMC1 [8]. Both recombinases polymerize on 3’ ssDNA overhangs to form nucleoprotein filaments that can be cytologically observed as foci on chromosomes [9,10]. At this step, meiotic DSB repair encounters two possibilities to repair DSB by HR, either using the sister chromatid (inter-sister recombination) or using the homologous chromosomes (inter-homolog recombination).

The invasion and strand exchange of ssDNA displaces one strand of the template DNA, resulting in a three-stranded joint molecule (d-loops). D-loops are precursors for different pathways leading to either reciprocal exchange (CO) or non-reciprocal exchange (non-crossovers) between the homologous chromosomes. Two pathways of CO formation, classified as class I and class II, have been characterized, with variable relative importance in different species [3]. Class I COs are dependent on the activity of a group of proteins collectively called ZMM (for Zip1-4, Msh4-5, Mer3) [11], which stabilize D-loop intermediates to promote formation of the double-Holliday junction intermediates [12]. MLH1 and MLH3 in conjunction with EXO1 promote the resolution of double-Holliday junctions as class I COs [13,14]. The formation of a Class I CO reduces the probability of another CO forming in the vicinity, a phenomenon termed as CO interference [15]. Additionally, recombination intermediates can be resolved by structure specific endonucleases including MUS81, producing class II COs, which are not subjected to interference [16–18]. In Arabidopsis, class I COs constitute 85–90% of COs, while remaining minority are class II COs [19][20]. Like in most eukaryotes, DSBs largely outnumber COs in Arabidopsis [21]. This suggests that active mechanisms prevent DSBs from becoming CO. Accordingly, several anti-CO factors are identified in different species [10,22–31].

Previously, our forward genetic screen identified FIDGETIN-LIKE-1 (FIGL1) as a negative regulator of meiotic COs in Arabidopsis [10]. Mutation in Arabidopsis *FIGL1* increases meiotic CO frequency by 1.8-fold compared to wild type and modifies the number and/or dynamics of RAD51/DMC1 foci. FIGL1 is widely conserved and is required for efficient HR in human somatic cells through a direct interaction with RAD51 [32]. Altogether, this suggests that FIGL1 is a conserved regulator of the strand invasion step of recombination, both in somatic and meiotic cells. FIGL1 belongs to the large family of AAA-ATPase proteins that are implicated in structural remodeling, unfolding and disassembly of proteins and oligomer complexes [33,34].

Here, we identified a new factor limiting COs in Arabidopsis that interacts directly with FIGL1, which we named FIDGETIN-LIKE-1 INTERACTING PROTEIN (FLIP). FLIP and its interaction with FIGL1 are conserved from plants to mammals, suggesting that the complex was present at the root of the eukaryotic tree. While this manuscript was under evaluation, the homologue of FLIP in rice (MEICA) was also shown to regulate meiotic recombination [35]. We further showed that *FLIP* and *FIGL1* act in the same pathway to negatively regulate meiotic CO formation, which appears to act on the regulation of the recombinases DMC1 and RAD51. Finally, we showed that both Arabidopsis and human FIGL1-FLIP complexes interact with both RAD51 and DMC1. Overall, this study identified a novel conserved protein complex that regulates a crucial step of homologous recombination.

## Results

### Identification of FIDGETIN-LIKE-1 Interacting protein (FLIP), an evolutionarily conserved partner of FIGL1

**Fig 1:**
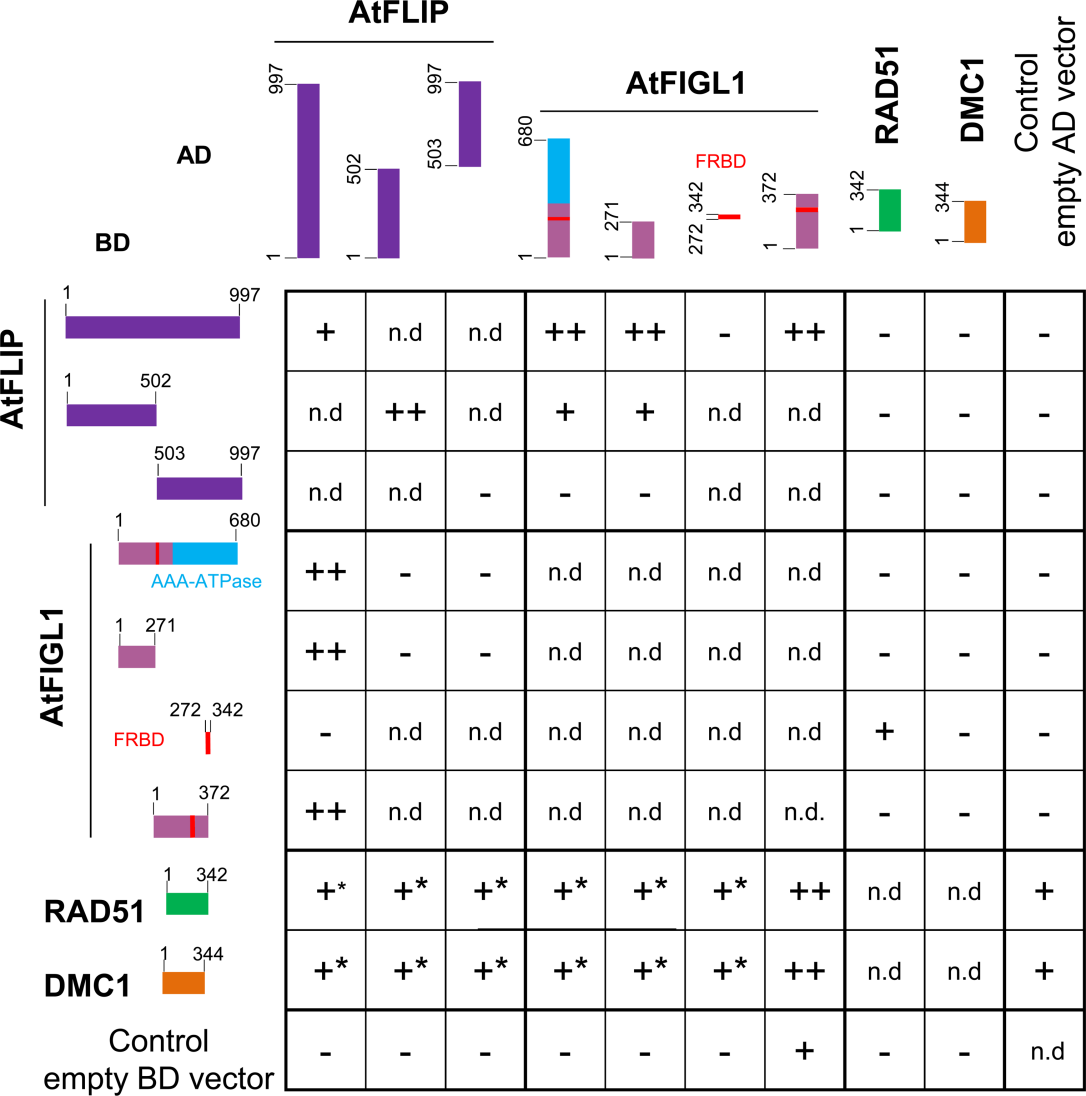
Yeast-two-hybrid experiments testing interactions between Arabidopsis FIGL1, FLIP, RAD51 and DMC1 proteins. Proteins of interest were fused with Gal4 DNA binding domain (BD, left) and with Gal4 activation domain (AD, top), respectively, and co-expressed in yeast cells. Full-length and truncated protein are schematically represented. For each combination, serial dilutions of yeast cells were spotted on non-selective medium (-LW), moderately selective media (-LWH) and more selective media (-LWHA). ++: Growth on both LWH and LWHA, interpreted as strong interaction. +: Growth on LWH and not on LWHA, interpreted as weak interaction. +*: Growth on LWH but cannot be interpreted as positive interaction because of auto-activation of one of the construct.—: Growth on neither LWH nor LWHA. n.d. Not determined. Pictures of yeasts are shown in S1 Fig.

We previously identified FIDGETIN-LIKE-1 (FIGL1) as an anti-CO protein [10]. To better understand the role of FIGL1 during meiotic recombination, we searched for its interacting partners by tandem affinity purification coupled to mass spectrometry (TAP-MS) using overexpressed FIGL1 as a bait in *Arabidopsis* suspension culture cells [36] (Table 1). After filtering co-purified proteins for false positives (see Materials and methods and [36]), we recovered, in two independent experiments, peptides from FIGL1 itself and a single additional protein. This single interacting protein is encoded by a gene of unknown function (*AT1G04650*), and we therefore named it as FIDGETIN-LIKE-1 INTERACTING PROTEIN (FLIP). Reciprocal TAP-MS experiments using FLIP as bait recovered only FLIP and FIGL1 peptides, further suggesting that FLIP and FIGL1 belong to the same complex *in vivo* (Table 1). Direct interaction between FLIP and FIGL1 was further supported by yeast two hybrid (Y2H) assay using full length proteins (Fig 1). To map the interaction domains, we truncated FIGL1 and FLIP proteins and tested their interaction in Y2H assays (Fig 1). The N-terminal region of FIGL1 (1–271 amino acids), which lacked both the AAA-ATPase domain and the sequence similar to the human FIGNL1’s RAD51 binding domain (FRBD), was sufficient to mediate the interaction with FLIP. Conversely, the N-terminal half of FLIP (1–502 aminoacids) was sufficient to mediate an interaction with FIGL1.

**Table 1:** Tandem affinity purification using FIGL1 and FLIP as baits. Two replicates of Tandem affinity purifications (TAP1 and TAP2) followed by mass spectrometry were performed using either FIGL1 (A) or FLIP (B) as a bait over-expressed in cultured cells. For filtering specific and false positive interactors, refer to Materials and Methods and [36]. The number of peptides and the fraction of the protein covered are indicated for each hit. Raw data are presented in S1 Table.

A	**Bait = FIGL1**	**TAP 1**		**TAP 2**	
	**protein name**	**Number of peptides**	**protein coverage %**	**Number of peptides**	**protein coverage %**
	FIGL1	47	70,5	42	62,3
	FLIP	34	39,4	30	36,8
B	**Bait = FLIP**	**TAP 1**		**TAP 2**	
	**protein name**	**Number of peptides**	**protein coverage %**	**Number of peptides**	**protein coverage %**
	FLIP	33	40,6	37	46,2
	FIGL1	18	33,2	22	40,5

**Fig 2:**
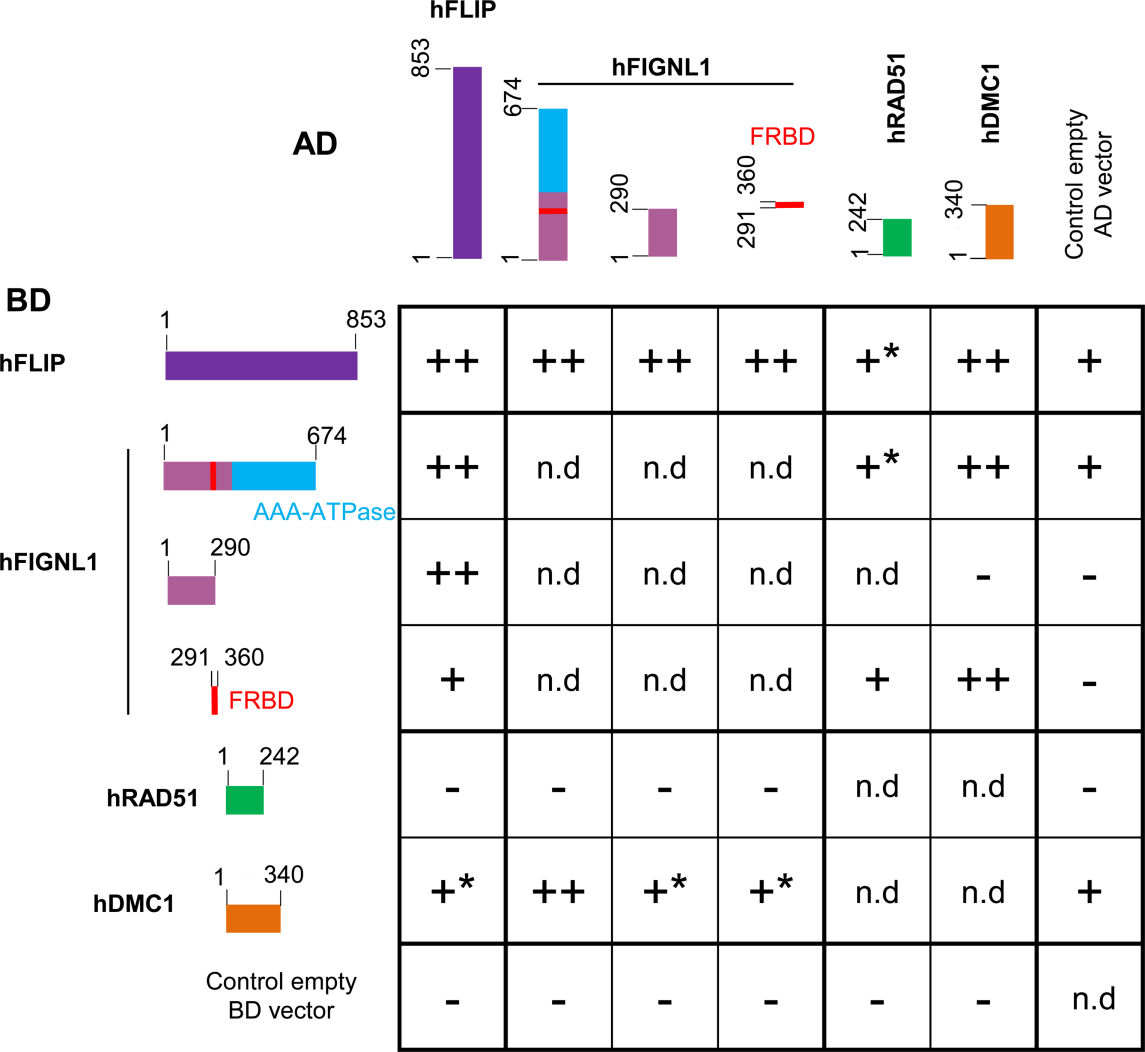
Yeast-two-hybrid experiments testing interactions between human FIGNL1, FLIP, RAD51 and DMC1 proteins. Proteins of interest were fused with Gal4 DNA binding domain (BD, left) and with Gal4 activation domain (AD, top), respectively, and co-expressed in yeast cells. Full-length and truncated protein are schematically represented. For each combination, serial dilutions of yeast cells were spotted on non-selective medium (-LW), moderately selective media (-LWH) and more selective media (-LWHA). ++: Growth on both LWH and LWHA, interpreted as strong interaction. +: Growth on LWH and not on LWHA, interpreted as weak interaction. +*: Growth on LWH but cannot be interpreted as positive interaction because of auto-activation of one of the construct. Growth on neither LWH nor LWHA. n.d. Not determined. Pictures of yeasts are shown in S2 Fig.

Further, the full length or the N terminal half of FLIP was able to interact with itself, suggesting that it could oligomerize (Fig 1). Moreover, the human orthologs of FLIP (C1ORF112, hFLIP) and FIGL1 (hFIGNL1) also showed interaction in our Y2H assays, suggesting that this interaction is evolutionarily conserved (Fig 2). hFIGNL1 and C1ORF112/hFLIP proteins were previously showed to co-purify in pull-down assays [32,37] and the mouse corresponding genes are strongly co-expressed [38], further supporting the conservation of the FIGL1-FLIP interaction from plants to mammals. The N-terminal region (1–290 aminoacids) of hFIGNL1, lacking the AAA-ATPase domain and the FRBD, was able to mediate the interaction with hFLIP, consistent with the Arabidopsis data (Figs 1 and 2). In addition, the FRBD of hFIGNL1 showed an interaction with hFLIP, suggesting that the FRBD domain could also contribute to the interaction (Fig 2). Finally, hFLIP was able to interact with itself, as shown for the Arabidopsis FLIP (Figs 1 and 2).

**Fig 3:**
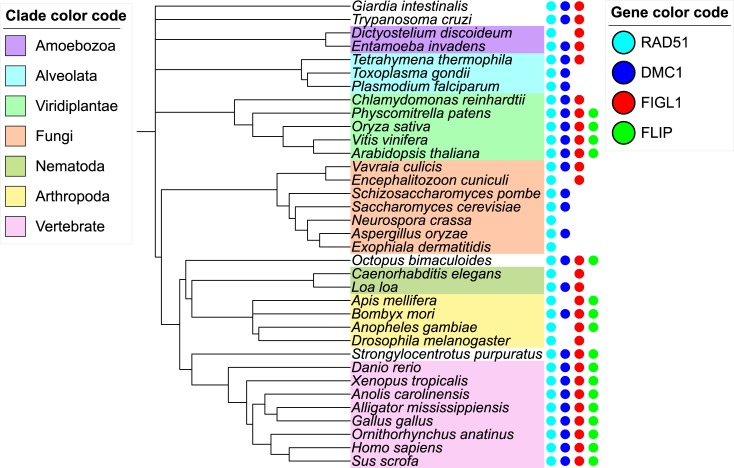
Phylogenetic tree depicting the evolutionary conservation of FLIP, FIGL1, RAD51 and DMC1 orthologs in a range of eukaryotic species. FLIP, FIGL1, DMC1 and RAD51 are presented as dots in green, red, blue and turquoise color, respectively. Gene accession numbers are provided in S2 Table. A version of this figure with a larger number of species can be found in S3 Fig and as an interactive tree at http://itol.embl.de/tree/132166555992271498216301.

The distribution of FLIP orthologs in eukaryotic species was analyzed using remote homology search strategy (see Methods). Orthologs of FLIP could be unambiguously detected in a wide range of species including mammalia, sauria and plants but also in arthropods and unicellular species such as choanoflagellate (Fig 3, S3 Fig for a larger number of species, and as interactive tree http://itol.embl.de/tree/132166555992271498216301). The FLIP orthologs showed low conservation at the sequence level (e.g. AtFLIP and hFLIP sharing only 12% sequence identity), but they all harbor a specific DUF4487 domain (Domain of Unknown Function) [39], further supporting their orthology. No FLIP ortholog could be detected in alveolata, amoebozoa and fungi. FLIP systematically co-occur with FIGL1, which is consistent with FLIP supporting the function of FIGL1 (Fig 3, S3 Fig). The reverse is not true since there are a number of species with FIGL1 ortholog detected but no FLIP (as in *D*. *melanogaster* and *C*. *elegans*). Structural predictions using RaptorX server[40] and HHpred [41] do not converge towards the same predicted fold but are both in agreement with FLIP likely folding as a long helical bundle over its full sequence. Such folds are often seen in protein recognition scaffolds suggesting FLIP could act as a FIGL1 adaptor module. Given the wide range of species harboring both FLIP and FIGL1 orthologs, the origin of this complex is probably quite ancient at the root of the eukaryotic tree suggesting that absence of FLIP-FIGL1 in some eukaryotic clades (such as Dikarya that regroups the fungi Basidiomycetes and Ascomycetes) is due to independent gene loss events.

### A genetic screen identified FLIP as an anti-CO factor

**Fig 4:**
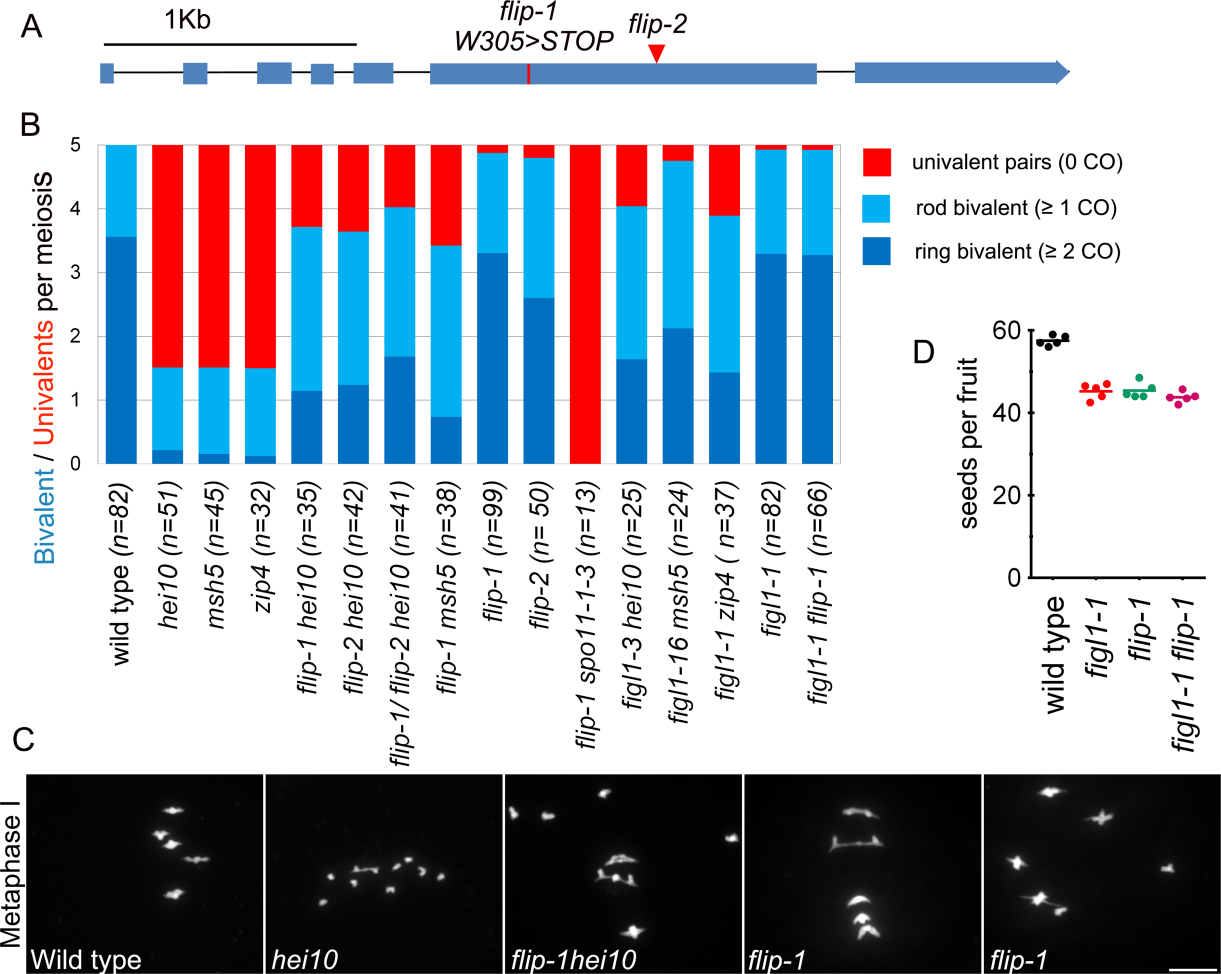
Mutation in *FLIP* restores crossover formation in *zmm* mutants: A. Schematic representation of the *FLIP* gene (Fidgetin-Like-1 Interacting Protein). Exons appear as blue boxes. The red line and red triangle indicate the missense mutation in *flip-1* and the *flip-2* T-DNA insertion, respectively. B. Average number of bivalents (blue) and pairs of univalents (red) per male meiocyte at metaphase I (Fig 4C). Light blue represents rod shaped bivalents indicating that one chromosome arm has at least one CO, and one arm has no CO. Dark blue represents ring shaped bivalent indicating the presence of at least one CO on both chromosome arms. The number of cells analyzed for each genotype is indicated in brackets. C. DAPI staining of Chromosome spreads of male meiocytes at metaphase I. Scale bars 10μm. D. Fertility measured as number of seeds per fruit. Each dot represents a plant; at least 10 fruits per plant were analyzed.

In parallel to the protein complex purification approach, *FLIP* was independently recovered in a genetic screen aiming at identifying meiotic anti-CO factors that previously uncovered *FIGL1*. Using fertility (fruit length) as a proxy for CO formation, we screened for ethyl methane sulfonate-generated mutations that restored COs in class I CO deficient mutants (*zmm*). As COs provide a physical link between pairs of chromosomes (bivalents), mutation of an anti-CO factor is expected to restore bivalent formation in CO-deficient mutants, thus improving balanced chromosome segregation and fertility [22]. This genetic screen led to the identification of several anti-CO factors, defining three pathways that limit COs in Arabidopsis:(i) The FANCM helicase and its cofactors [22,23]; (ii) The AAA-ATPase FIDGETIN-LIKE-1 (FIGL1) [10]; (iii) The RECQ4 helicase-Topoisomerase 3α-RMI1 complex [24,25]. Here, we isolated an additional suppressor of *hei10*, one of the *zmm* mutants that are deficient in class I COs [42]. This suppressor, *hei10(S)320* showed longer fruit length compared to *hei10* and bivalent formation was restored to an average of 3.7 bivalents per cell compared to 1.5 in *hei10* and 5 in wild type (Fig 4), suggesting a partial restoration of CO formation. Whole genome sequencing and genetic mapping of *hei10(S)320* defined a genetic interval containing five putative causal mutations. One of them resulted in a stop codon in the gene *AT1G04650*, which encodes FLIP (*flip-1* W305>STOP) (Fig 4). An independent mutation in *FLIP* (T-DNA Salk_037387/ *flip-2*), was also able to restore bivalent formation in *hei10* (Fig 4). Further, *flip-1/flip-2 hei10* exhibited restored bivalents (Fig 4), demonstrating that *flip-1* and *flip-2* are allelic and that mutations in *FLIP* are causal for the restoration of bivalents in *hei10*. The *flip-1* mutation was also able to restore bivalent formation in *msh5* (Fig 4), another essential gene of the class I CO pathway, suggesting that effect of the *flip* mutation is not specific to *hei10* but allows the formation of COs in absence of the class I pathway.

No growth or development defects were observed in the *flip* mutants. Meiosis progressed normally in single *flip-1* and *flip-2*, except that a pair of univalent was observed at metaphase in ~14% of the cells (n = 12/99 in *flip-1*; n = 9/50 in *flip-2)*. (Fig 4B and 4C). Similarly, we observed a low frequency of univalents in *figl1-1*(n = 6/82 cells) that has been missed in previous analyses [10], and in *figl1-1 flip-1* (n = 5/66 cells). This suggests a slight defect in implementation of the obligate COs in absence of *FLIP or FIGL1*. We also observed a moderate increase in the frequency of pollen death (wild type 1.1% ±0.3, *figl1-1* 5.2% ±1.8, *flip-1* 5.8% ±0.6, *figl1-1 flip-1* 4.3% ±1.1; n = 5 plants per genotype, ≥300 pollen grains/plant) and a decrease in the number of seeds per fruit was observed in the single and double mutants (Fig 4D).

**Fig 5:**
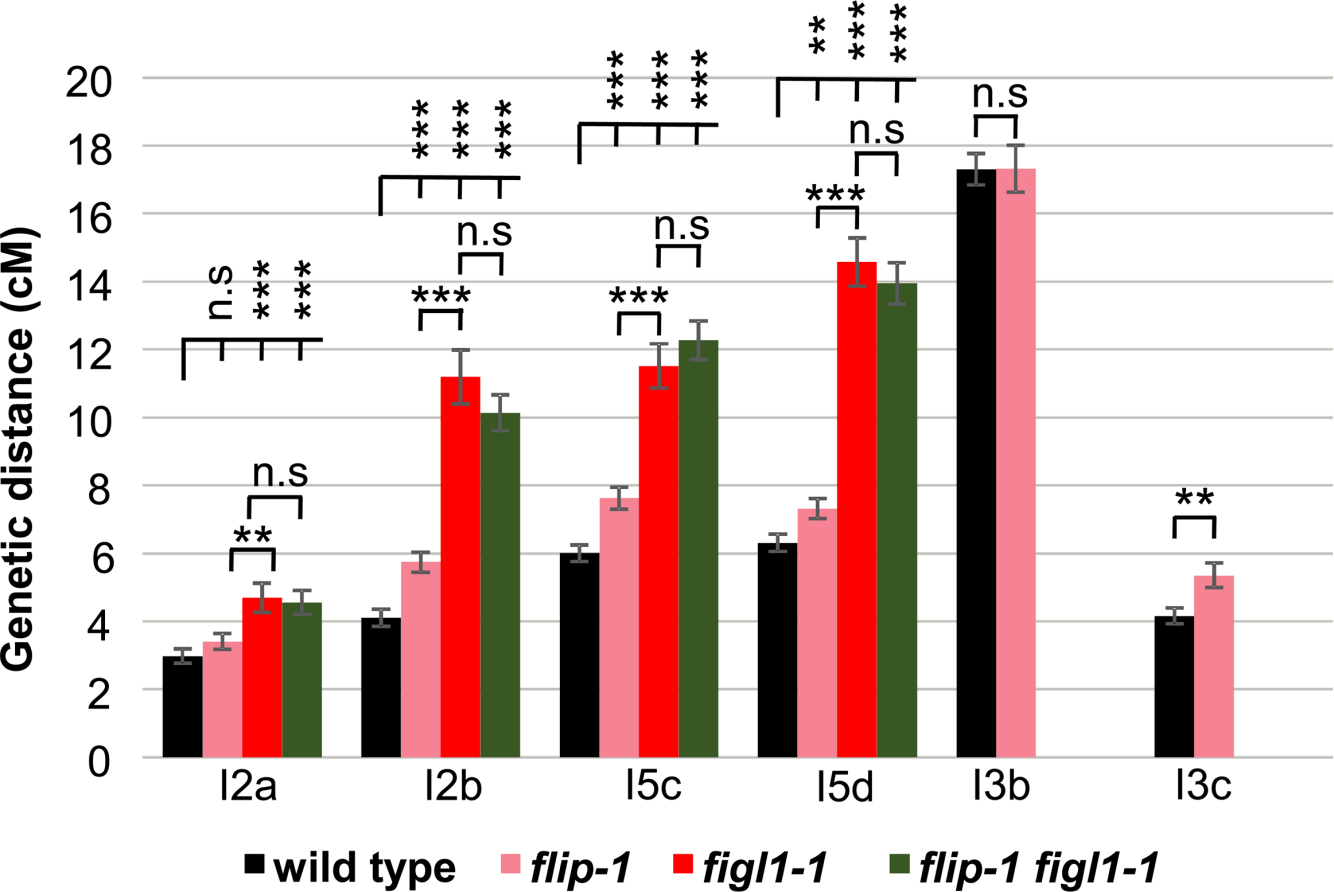
*FLIP* and *FIGL1* act in the same pathway to limit COs. Genetic distance in centiMorgan (cM) measured by pollen tetrad analysis using fluorescent tagged lines [43]. I2a and I2b are adjacent intervals on chromosome 2. Similarly I3bc and I5cd on chromosome 3 and 5, respectively. Error bar indicates ± standard error of the mean. Not significant (n.s) *p* > 0.05; ** *p* < 0.01; *** *p* < 0.001, Z-test. Raw data are presented in S3 Table.

We next monitored the direct effect of *FLIP* mutation on CO frequency by tetrad analysis and measured recombination in six genetic intervals defined by fluorescent tagged markers that confer fluorescence in pollens [43]. CO frequencies in *flip-1* were significantly increased in four intervals out of six tested, in the range of +15% to +40% compared to wild type (Fig 5). This increase in CO frequencies due to loss of *FLIP* is consistent with the restoration of bivalent formation in *zmm* mutants and implies that FLIP limits COs during meiosis in wild type. FLIP physically interacts with FIGL1 (see above), suggesting that they can act together to limit COs. We therefore compared recombination in *flip-1*, *figl1-1* and the double mutant by tetrad analysis. On the four intervals tested, *figl1-1* showed an average of ~70% CO increase compared to wild type, corroborating previous findings (Fig 5), which is significantly higher than *flip-1*. Combining *flip-1* and *figl1-1* mutations did not lead to a further increase in recombination suggesting that *FIGL1* and *FLIP* act in the same pathway to negatively regulate CO formation (Fig 5). However, FIGL1 may be partially active in absence of FLIP as *flip-1* increases CO frequencies to a lesser extent than *figl1-1*.

### *FLIP* limits class II COs

**Fig 6:**
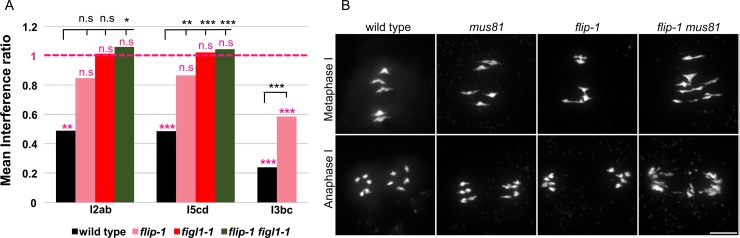
*FLIP* limits Class II COs. A. Interference ratio is the ratio of the genetic size in an interval with CO in an adjacent interval divided by the genetic size of the same interval without CO in the adjacent interval. This ratio provides an estimate of the strength of CO interference. IR close to 0 means strong interference; Interference ratio = 1 (purple line) indicates that interference is absent. The test of absence of interference is shown in purple (n.s *p* > 0.05; ** *p* < 0.01; *** *p* < 0.001). Comparison of Interference ratio between the genotypes wild type and mutants is indicated in black (n.s *p* > 0.05; * *p* < 0.05 ** *p* < 0.01; *** *p* < 0.001). B. Chromosome spreads of male meiocytes at metaphase I and anaphase I. Scale bars 10μm.

We next explored the origin of extra COs in *flip*. In the *flip-1 spo11-1* double mutant, bivalent were completely abolished and 10 univalents were observed at metaphase I, (Fig 4B), showing that all COs in *flip-1* are dependent on SPO11-1 induced DSBs. Two classes of COs exist in *Arabidopsis*: class I COs are dependent on ZMM proteins and are subjected to interference, while class II are insensitive to interference and involve structure specific endonucleases including MUS81 [21]. The *flip-1* mutation restored CO formation in two *zmm* mutants, *hei10* and *msh5* (see above). Further, tetrad analysis of three pairs of intervals showed reduced interference in *flip-1* compared to wild type (Fig 6A). Finally, we examined meiosis in the *flip-1 mus81* double mutant. While no chromosome fragmentation is observed in single *flip-1* or *mus81* mutants, chromosome fragments were observed at anaphase I in the *flip-1 mus81* double mutant (n = 31/31 cells. Fig 6B). This suggests that MUS81 is required for resolution of recombination intermediates formed in *flip-1*. Altogether, the extra COs produced in *flip-1* appeared to be dependent on the class II pathway, as previously shown for *figl1-1* [10].

### FIGL1 and FLIP regulate RAD51 and DMC1 focus dynamics

**Fig 7:**
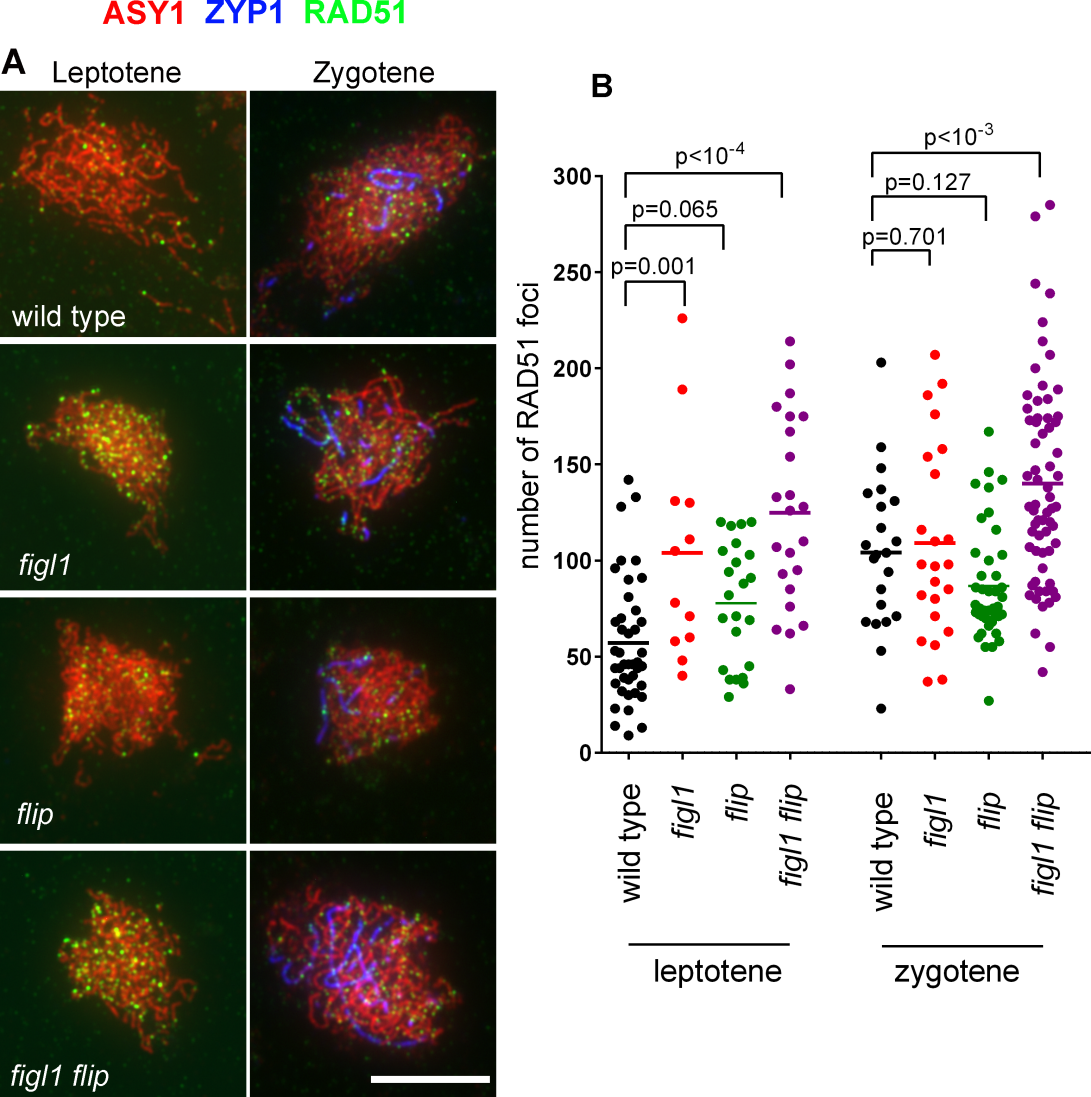
RAD51 foci in wild type, *figl1*, *flip* and *figl1 flip*. A. Triple immunolocalization of ASY1 (red), ZYP1 (blue) and RAD51 (green) on meiotic chromosome spreads. Merged pictures are shown. Partial ZYP1 polymerization defines the zygotene stage. Scale bars 10μm. B. Quantification of RAD51 foci at leptotene and zygotene in wild type, *figl1*, *flip* and *figl1 flip*. Each dot represents an individual cell and bars indicate the mean. P values are the results of Fisher's LSD tests.

**Fig 8:**
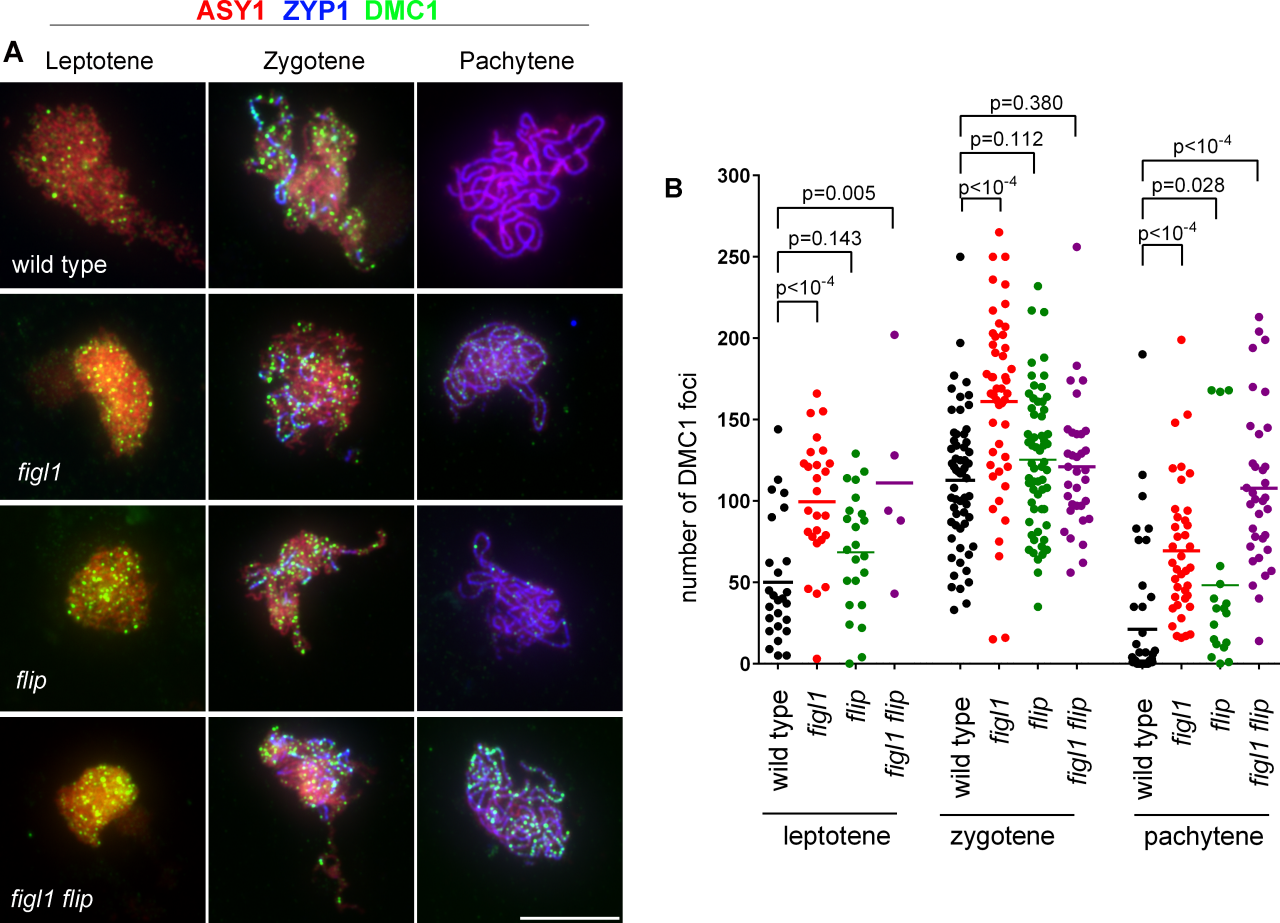
DMC1 foci in wild type *figl1*, *flip* and *figl1 flip*. A. Triple immunolocalization of ASY1 (red), ZYP1 (blue) and DMC1 (green) on meiotic chromosome spreads. Merged pictures are shown. Partial and full ZYP1 polymerization defines the zygotene and pachytene stages, respectively. Scale bars 10μm. B. Quantification of DMC1 foci at leptotene, zygotene and pachytene in wild type, *figl1*, *flip* and *figl1 flip*. Each dot represents an individual cell and bars indicate the mean. P values are the results of Fisher's LSD tests.

Based on genetic and physical interactions between FIGL1 and FLIP, we next hypothesized that FLIP might regulate RAD51 and DMC1 foci during meiosis, as previously shown for FIGL1 [10]. We thus performed RAD51 (Fig 7) and DMC1 (Fig 8) immunolocalization in *figl1*, *flip* and *figl1 flip* in combination with staining of the chromosome axis (ASY1) and the synaptonemal complex (ZYP1) to follow their localization at early, mid and late prophase stages.

In wild type, RAD51 foci appear at leptotene and increase at zygotene (Fig 7). The number of RAD51 foci at leptotene is increased by ~2 fold in *figl1 and figl1 flip*. An increase is also observed in *flip* at leptotene, but to a lesser extent and at the edge of significance. At zygotene the number of RAD51 foci was not significantly different between the two single mutants and the wild type, but appeared increased in *figl1 flip*. This suggests that FIGL1/FLIP negatively regulates the formation or the turnover of RAD51 foci.

In wild-type, DMC1 foci first appear at leptotene, peak at zygotene and almost disappear at pachytene (33/46 had less than 10 foci) (Fig 8). At both leptotene and pachytene, a large increase of DMC1 foci was observed in *figl1* and *figl1 flip*. The same trend was observed in *flip*, but with a lesser increase and barely significant. At zygotene, only the single *figl1* had a significantly higher number of DMC1 foci. Altogether, this suggests that FIGL1/FLIP regulate the kinetics of appearance and disappearance of DMC1 foci, with FIGL1 playing a more central role than FLIP. Persistence of DMC1 foci may represent unrepaired DSBs that are eventually repaired (possibly by MUS81), as no chromosome fragmentation was observed at anaphase I in *figl1* or *flip* mutant.

**Fig 9:**
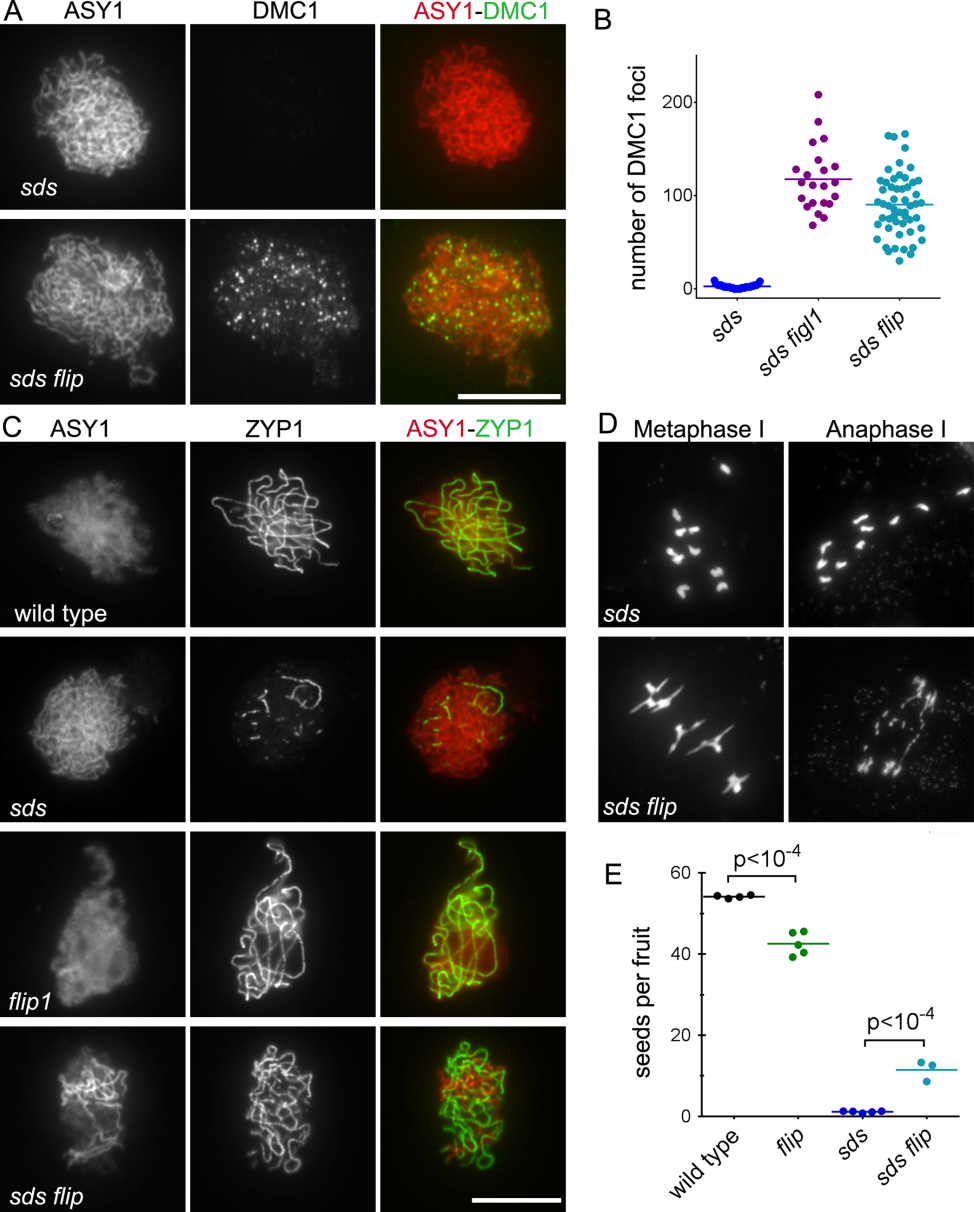
*FLIP* genetically interacts with *SDS*. A. Immunostaining of DMC1 (green) and the chromosome axis protein ASY1 (red) on leptotene/zygotene meiotic chromosome spreads. B. Quantification of DMC1 foci at leptotene/zygotene in *sds*, *sds figl1* and *sds flip*. Each dot represents an individual cell and bars indicate the mean. C. Co-immunolocalization of ASY1 (red) and ZYP1 (green), which mark respectively chromosome axes and synapsed regions. Synapsis was partially restored in *sds flip* compared to single mutant *sds*. Scale bars 10μm. D. DAPI staining of chromosome spreads of male meiocytes at metaphase I and anaphase I. Scale bars 10μm. E. Fertility measured as number of seeds per fruit. Each dot represents a plant; at least 12 fruits per plant were analyzed. P values are the results of Fisher's LSD tests.

One known positive regulator of DMC1 in plants is SDS, a meiosis-specific cyclin-like protein [44,45]. In absence of SDS, DMC1 foci do not form, synapsis and COs are abolished, but DSBs and RAD51 foci are formed and repair is completed, presumably using the sister as template [44,45]. We previously showed that mutation in *FIGL1* restores DMC1 focus formation, synapsis, and bivalent formation in *sds* [10]. These results argued for antagonistic functions of SDS and FIGL1, the former positively and the latter negatively regulating DMC1 foci formation and DMC1-mediated homolog engagement. Here, we similarly showed that DMC1 foci and synapsis are partially restored in *sds flip* double mutants as compared to *sds* (Fig 9A, 9B and 9C). Moreover, 4 to 5 bivalents per metaphase I were observed in *sds flip* (n = 57) while their formation is almost completely abolished in *sds* (0.12 bivalents per metaphase I, n = 50) (Fig 9D). However, recombination is not completely restored in *sds flip* as chromosome fragmentation is observed at anaphase I. Accordingly, fertility is only partially restored in *sds flip* compared to *sds* (Fig 9E). Taken together, this strongly suggests that FIGL1 and FLIP antagonize SDS in the regulation of DMC1 focus formation and DMC1 mediated inter-homolog interactions and crossover formation. In both *figl1 sds* [10] and *sds flip* (Fig 9D), bivalents at metaphase I had slightly aberrant shape and chromosome fragmentation was observed at anaphase I. This suggests that FIGL1 and FLIP may have a function in DSB repair downstream of homologous template invasion or that the recombination intermediates restored in absence of both *sds* and *figl1/flip* are aberrant.

### The FLIP-FIGL1 complex interacts with RAD51 and DMC1

Our genetic interaction and immuno-localization studies in Arabidopsis suggest that the FIGL1/FLIP complex might regulate the function of RAD51 and DMC1, directly or indirectly. In addition, it was shown that human FIGNL1 interacts with human RAD51 through a domain called FRBD [32]. Hence, we set out to examine whether Arabidopsis and human FIGL1 and FLIP interact with RAD51 and DMC1, using Y2H assays. Consistent with published data, the Y2H assay detected an interaction between the FRBD domain of human FIGNL1 and RAD51, though it was weak and only positive in one direction (Fig 2). Similarly, we detected an interaction between Arabidopsis FIGL1 and RAD51, mediated by the predicted FRBD domain (Fig 1). In addition, we observed a clear interaction between human FIGNL1 and DMC1, mediated by the FRDB domain (Fig 2). Arabidopsis FIGL1 interacted also with DMC1, although the interaction was detected only in one direction (Fig 1). This suggests that FIGL1 can interact directly with both RAD51 and DMC1 and that these interactions are conserved in plants and mammals.

Next, we tested interaction between FLIP and the two recombinases, with both plant and human proteins. Human FLIP interacted with DMC1, suggesting that FLIP could reinforce the interaction of the FIGL1-FLIP complex with DMC1 (Fig 2). However, our Y2H assay did not reveal any interaction between Arabidopsis FLIP and DMC1 (Fig 1). No interaction was detected between FLIP and RAD51, for both human and Arabidopsis proteins (Figs 1 and 2).

## Discussion

We identified, by two different approaches, FLIP as a new factor that genetically and physically interacts with FIGL1 [10] and regulates meiotic recombination. We showed that (i) FIGL1 and FLIP form a conserved complex; (ii) *FLIP* and *FIGL1* are anti-CO factors that act in the same pathway to regulate meiotic recombination; (iii) kinetics of DMC1 and RAD51 foci are modified in *figl1* and, to a lesser extent, in *flip*; (iv) *flip* and *figl1* restore DMC1 focus formation and inter-homolog interactions (synapsis) in the *sds* mutant; (v) FIGL1-FLIP complex interacts with RAD51 and DMC1, and this interaction is evolutionarily conserved in both plants and mammals. FIGL1 was previously shown to be involved in meiotic recombination in Arabidopsis, and in recombination-mediated DNA repair in human somatic cells [10,32,46]. In contrast and despite the conservation in many eukaryotes, FLIP was of unknown function. We propose a model wherein FIGL1 and FLIP act as a complex that negatively regulates the strand invasion step of HR by interacting with DMC1/RAD51 and modulating their activity/dynamics. FIGL1 belongs to the AAA-ATPase group of proteins, which typically function by dismantling the native folding of their target proteins [33,34]. Therefore, it is tempting to suggest that the FLIP-FIGL1 complex may directly disrupt DMC1/RAD51 filaments using the unfoldase activity of FIGL1. Supporting this possibility, both Arabidopsis and human FIGL1 physically interact with DMC1 and RAD51.

We showed that FLIP and FIGL1 act together to limit meiotic COs in Arabidopsis, but the increase in CO frequency is lower in *flip* than in *figl1* (~30% and ~70% increase compared to wild type, respectively). This difference in CO frequency could be attributed to the catalytic activity of the complex being supported by FIGL1. We suggest that FLIP could only be partially required for FIGL1 enzymatic functions *in vivo*, acting as a co-factor or reinforcing the affinity and/or the specificity of the interaction of the FIGL1/FLIP complex with the target. In our assay, human FLIP interacted with DMC1, suggesting that FLIP could indeed function to facilitate FIGL1 activity towards DMC1. We could not detect an interaction between FLIP and RAD51 but we cannot rule out the possibility that FLIP facilitates also interaction of the complex with RAD51. Indeed, several lines of evidence suggest that FLIP could act in conjunction with FIGL1 in its role in somatic HR [32]: Down-regulation of *hFLIP* induces reduced growth of HeLa cells [38]. *FLIP* in mouse is strongly co-expressed with cancer related genes and the knock out mouse is not viable [38,47]. Finally, *FIGNL1* and *hFLIP* are strongly co-regulated in mouse expression data [38]. Overall, this argues for a conserved role of the FIGL1/FLIP complex in regulating RAD51/DMC1 activities during both somatic and meiotic HR.

Beyond Arabidopsis and humans, *FIGL1* and *FLIP* are conserved in all vertebrates and land plants examined in the current study. FIGL1 and FLIP can be also detected in species from other distant clades, suggesting that this complex emerged early in the evolution of eukaryotes (Fig 3). However, some clades appear to have lost both *FIGL1* and *FLIP*, most notably the *Alveolata* and *Dikarya* (which regroups the fungi *Basidiomycetes* and *Ascomycetes*). In those species, RAD51/DMC1 might be regulated independently of FIGL1-FLIP. Species with a *FLIP* ortholog also systematically have a *FIGL1*, but the reverse is not true, several species/clades having *FIGL1* but no detectable *FLIP* orthologs. This is consistent with our experimental data that argue for FIGL1 being the core activity of the complex and FLIP as a dispensable factor for FIGL1 activity. While *RAD51* appears to be universally conserved, *DMC1* is absent in a number of species (Fig 3). Moreover, we could not find any correlation between presence/absence of *FIGL1* or *FLIP* with *DMC1*. Some species have *DMC1* but no *FIGL1/FLIP* (e.g. many fungi), while others have *DMC1* and *FIGL1* but not *FLIP* (e;g some nematodes), or *FIGL1* and *FLIP* without *DMC1* (e.g. *Chrophyta*). Altogether, our phylogenic analysis supports that neither FIGL1 nor FLIP are specific to DMC1, and that the FIGL1-FLIP complex can regulate the activity of both RAD51 and DMC1. The FIGL1 complex may also have additional functions unrelated to HR [48].

We suggest that FIGL1 and FLIP could limit strand invasion mediated by RAD51 and DMC1. How could the lack of this function lead to an increase in the frequency of meiotic COs as observed in *flip* and *figl1*? One conceivable explanation is that the absence of FLIP and FIGL1 changes the equilibrium between invasions on inter-sister versus inter-homolog, leading to the formation of higher numbers of inter-homolog joint molecules and eventually more COs. However, DSBs and presumably inter-homologous joint molecules are already in large excess to COs in wild type [21], making it hard to believe that a simple increase in their number would increase CO frequencies. We favor another possibility in which the lack of the FLIP / FIGL1 activity generates aberrant recombination intermediates through either multi-chromatid invasions or invasion of both ends of a break. The observation that the structure specific nuclease MUS81 becomes essential for completion of repair in *figl1* and *flip* suggests that indeed some novel class of intermediates arise in these mutants. Thus, we favor the hypothesis in which the absence of *FLIP* and *FIGL1* leads to excessive and/or late activity of DMC1/RAD51, generating aberrant joint molecules such as multi-chromatid joint molecules [49,50]. Such unusual structures would need structure specific endonucleases to be resolved, leading to increased COs. Therefore, the function of FLIP-FIGL1 in wild type context could prevent formation of aberrant recombination intermediates by functioning as a quality control of strand invasion.

Intriguingly, some univalents are observed at metaphase in *figl1* and *flip*. This suggests that the implementation of the obligate CO is slightly affected in absence of FIGL1/FLIP. One possibility is that some recombination intermediates designated to become COs fail to mature into actual COs because they have aberrant structures generated by unregulated DMC1/RAD51. In such scenario, these intermediates would be eventually repaired as non-crossovers, as no chromosome fragmentation is observed in the mutants.

While this manuscript was under evaluation, the homologue of *FLIP* in rice (*MEICA*) has been shown to regulate meiotic recombination [35]. The mutation of *meica* restores COs in *msh5*, suggesting that the anti-CO function of *FLIP/MEICA* is conserved in plants. However, both *Osfignl1* [51] and *meica* [35] mutants in rice show significant chromosome fragmentation at anaphase I, suggesting that the FIGL1-FLIP/Os FIGNL1-MEICA complex is more crucial for the completion of DSB repair in rice than in Arabidopsis.

In conclusion, we uncovered a conserved FIGL1-FLIP complex that directly binds to RAD51/DMC1 and could negatively regulate strand invasion during homologous recombination. It would be of particular interest to further study the function of this complex in mammalian systems and in biochemical assays. Unraveling proteins playing a role in HR pathway would provide better understanding related to various inherited diseases in humans pertaining to defects in HR repair proteins [2]. Targeting HR protein could increase the sensitivity of cancer cells to anti-cancer drugs [52]. Thus, FIGL1-FLIP could represent potential targets for cancer therapy.

## Materials and methods

### Genetic material

The *Arabidopsis* lines used in this study were: *hei10-2* (N514624) [42], *msh5-*2 (N526553) [53], *mus81-2* (N607515) [18], *spo11-1-3* (N646172)[54], *sds-2* (N806294)[44],*figl1-1* [10], *zip4-2* (N568052) [55]. Tetrad analysis lines (FTLs) used were as follows: I2ab (FTL1506/FTL1524/FTL965/*qrt1-2*), I3bc (FTL1500/FTL3115/FTL1371/*qrt1-2*) and I5cd (FTL1143/FTL1963/FTL2450/*qrt1-2*). FTLs were obtained from Gregory Copenhaver [43]. Suppressor *hei10(s)320/flip-1* was sequenced using iIlumina technology at the Genome Analysis Centre, Norwich, UK. Mutations were identified through MutDetect pipeline [23]. The *flip-1* causal mutation was C to T substitution at the position chr1:1297137 (Col-0 TAIR10 assembly). *flip-2* (N662136) T-DNA mutant was obtained from the Salk collection, distributed by the NASC. The primers used for genotyping are listed in the S4 Table.

### Cytology techniques

Meiotic chromosomes from anthers were spread and DAPI stained as previously described [56]. For cytological detection of meiotic proteins, male meiotic chromosome spreads from prophase I were prepared as described in Armstrong *et al*. [57]. Spread slides were either immediately used for immuno-cytology or stored at -80°C before immunostaining. Chromosome axis protein ASY1 and synaptonemal complex protein ZYP1 staining were performed to define substages of prophase I. Leptotene stage had only ASY1 signal, while zygotene and pachytene cells showed partial stretches of ZYP1 signal or 95–100% of ZYP1 signal in the nucleus, respectively. Primary antibodies used for immunostaining were: anti-DMC1 (1:20) [58], anti-RAD51 (1:500) [59], anti-ZYP1 raised in rat (1:250) [60] or rabbit (1:500) and anti-ASY1 raised in guinea pig (1:250) or chicken (1:50) [57]. Secondary antibody: Alexa fluor 488 (A-11006); Alexa fluor 568 (A-11077); Alexa fluor 647 (A-11006), anti-rabbit 647 (6444–31 Southern Biotech) and super clonal Alexa fluor®488, (A-27034) obtained from Thermo Fisher Scientific were used in 1:400 dilution. Images were obtained using a Zeiss AxioObserver microscope and were analyzed by Zeiss Zen software. In case of DMC1 and RAD51 staining, all images were acquired at 2s exposure, and DMC1 and RAD51 foci were counted by using Fiji software after exporting images in tiff format [61]. Briefly, DAPI or ASY1 images were binarized using the ‘triangle’ intensity thresholding method followed by a binary morphological closing operation to localize meiotic chromosomes and to mark them as regions of interest (ROI). In parallel, a white top-hat transform was applied to DMC1 or RAD51 images. Significant peaks located within chromosome ROI were counted as foci. Scatter dot plots and statistical analysis were performed using the software GraphPad Prism 6.

### Recombination measurement

We used FTLs [43] to estimate male meiotic recombination rates at three pairs of genetic intervals I2ab, I5cd and I3bc. For each set of experiment, heterozygous plants were generated for the pairs of linked fluorescent markers and siblings from the same segregating progeny were used to compare the recombination frequency between different genotypes. Slides were prepared as described previously [43]. Tetrads were counted and sorted to specific classes (A to L) [43] using a pipeline developed on the Metafer Slide Scanning Platform. For each tetrad, attribution to a specific class was double checked manually. Genetic sizes of each interval was calculated using Perkins equation [62] as follows: D = 100× (Tetratype frequency+6× Non-Parental Ditype frequency)/2 in cM. The Interference ratio (IR) was measured as described previously [63] [43]. Briefly, in two adjacent intervals I1 and I2, genetic size of I1 was calculated for the two populations of tetrads in I2 interval–D1 is at least with one CO in I2; D2 is without CO in I2. The ratio of D1/D2 revealed presence (when IR<1) or absence (when IR is close to 1 or >1) of the interference. A chi square test is performed to test the null hypothesis (H0: D1 = D2). The average of the two reciprocals is depicted on the graph (Fig 6A).

### Cloning

Cloning of the *FIGL1* open reading frame (ORF) is described in [10]. The *AtFLIP* ORF was amplified using gene-specific primer (S4 Table) on cDNA prepared from Arabidopsis flower buds (Col-0 accession). The full length or truncated ORFs of *FLIP* were cloned into pDONR207/pDONR201 vectors to produce entry clones. All plasmid inserts were verified by Sanger sequencing. The ORFs for human FIGNL1 (BC051867), RAD51 (BC001459), DMC1 (BC125163) were obtained from the human orfeome collection, while human FLIP (IMAGE clone: 30389801) ORF was ordered from Source BioScience, UK

### Yeast two hybrid assay

For yeast two hybrid assays, *AtFIGL1*, *AtFLIP*, *AtRAD51 and AtDMC1* as well as their respective human orthologs (*hFIGNL1*, *hFLIP*, *hRAD51*, *hDMC1*) were cloned into destination vectors pGBKT7 and pGADT7 by the Gateway technology. The fidelity of coding sequence of all clones was verified by sequencing. Yeast two hybrid assays were carried out using Gal4 based system (Clontech) [64] by introducing plasmids harboring gene of interest in yeast strains AH109 and Y187 and interaction were tested as previously described [65].

### Tandem affinity purification coupled with mass spectrometry (TAP-MS)

TAP-MS analysis was performed as described previously [36]. Briefly, the plasmids expressing FLIP or FIGL1 fused to the double affinity GS^rhino^ tag [36] were transformed into *Arabidopsis* (Ler) cell-suspension cultures. TAP purifications were performed with 200 mg of total protein extract as input and interacting proteins were identified by mass spectrometry using an LTQ Orbitrap Velos mass spectrometer. Proteins with at least two high-confidence peptides were retained only if reproducible in two experiments. Non-specific proteins were filtered out based on their frequency of occurrence in a large dataset of TAP experiments with many different and unrelated baits as described [36].

### Bioinformatics

Identification of putative orthologs of FLIP, FIGL1, DMC1 and RAD51 was performed following different strategies based on the sequence divergence and the existence of paralogs. Since FLIP sequence diverged significantly during evolution without detectable paralog, 3 iterations of HHblits [66,67] against the uniclust30_2017_04 database were sufficient to retrieve 139 sequences belonging to plants and metazoa species. To get NCBI entries of those proteins, a PSSM generated from the recovered alignment was used as input of a jump start PSI-blast [68] against the eukaryotic refseq_protein database [69]. For DMC1 and RAD51, reciprocal best hits of blast searches were used to identify the most likely ortholog in every species. First, DMC1 in *H*. *sapiens* and *S*. *cerevisiae* sequences were blasted against the refseq_protein database to gather a set of DMC1 candidates. Each of these candidates was reciprocally blasted against the protein sequences of six fully sequenced genomes wherein DMC1 and RAD51 genes could be unambiguously identified and which were chosen spread over the phylogenetic tree (*H*. *sapiens*, *S*. *cerevisiae*, *C*. *reinhardtii*, *T*. *gondii*, *P*. *falciparum*, *T*. *cruzi*). Detection of a DMC1 ortholog was considered correct when one of the 6 DMC1 genes was spotted out as best hit with an alignment score at least 10% higher than that of the second best hit, supporting its significantly higher similarity to DMC1 than to RAD51. The same strategy was followed to assign RAD51 orthologs. In the case of FIGL1, large number of paralogs such as spastin, fidgetin, katanin or sap1-like proteins render the global analysis more complex. A phylogenetic tree was initially built focused on the AAA ATPase domain of 600 protein sequences belonging to fidgetin, spastin, katanin, sap1 and VPS4 families. They were aligned using mafft einsi algorithm [70] and tree was built with PhyML [69] using the LG model for aminoacid substitution and 4 categories in the discrete gamma model. This prior analysis helped to delineate which homologs could be considered as orthologs of *H*. *sapiens* and *A*. *thaliana* FIDGETIN-like proteins. For the 373 fully sequenced species presented in Fig 3, reciprocal blast best hit searches were then performed to retrieve the Fidgetin-like ortholog when present. FIGL1 ortholog candidates were retrieved from a blast of *H*. *sapiens* and *A*. *thaliana* FIGL1 sequences against the refseq_protein database and were assessed by reciprocal best hit searches using these candidates as query against genomes of *H*. *sapiens* and *A*. *thaliana*. Detection of FIGL1 orthology was assessed if best hit was FIGL1 sequence with an alignment score at least 10% higher than that of the second best hit. For a limited number of species, orthologs were suspected but not identified in any of the NCBI databases. Targeted blast searches where then performed on their genomes using the Joint Genome Institute (JGI) server to further probe the existence of these orthologs which could be detected in 7 cases. All the NCBI and JGI gene entries are listed in S2 Table and can be easily retrieved from the interactive tree (http://itol.embl.de/tree/132166555992271498216301) [71] by passing the mouse over the species names.

## Supporting information

S1 Fig Yeast-two-hybrid experiments testing interactions between Arabidopsis FIGL1, FLIP, RAD51 and DMC1 proteins. Proteins of interest were fused with Gal4 DNA binding domain (BD) and with Gal4 activation domain (AD), respectively and co-expressed in yeast cells. For each combination, serial dilutions of yeast cells were spotted on non-selective medium (-LW), moderately selective media (-LWH) and more selective media (-LWHA). Growth on LWH is interpreted as weak interaction and growth on LWHA is interpreted as strong interaction.
(PDF)

S2 Fig Yeast-two-hybrid experiments testing interactions between human FIGNL1, FLIP, RAD51 and DMC1 proteins. Proteins of interest were fused with Gal4 DNA binding domain (BD) and with Gal4 activation domain (AD), respectively, and expressed in yeast cells. For each combination, serial dilutions of yeast cells were spotted on non-selective medium (-LW), moderately selective media (-LWH) and more selective media (-LWHA). Growth on LWH is interpreted as weak interaction and growth on LWHA is interpreted as strong interaction.
(PDF)

S3 Fig Phylogenetic tree depicting the evolutionary conservation of FLIP, FIGL1, RAD51 and DMC1 orthologs in a large range of eukaryotic species. All the NCBI and JGI gene entries are listed in S2 Table and can be retrieved from the interactive tree (http://itol.embl.de/tree/132166555992271498216301).
(PDF)

S1 Table TAP-MS data. (XLSX)

S2 Table NCBI and JGI gene entries of Figs 3 and S3. (XLSX)

S3 Table Raw FTL data. (XLSX)

S4 Table Genotyping primers. (XLSX)
