# Supplementary figures and images for: FIGL1 and its novel partner FLIP form a conserved complex that regulates homologous recombination
Source: PLoS Genet. 2018 Apr 2;14(4):e1007317. doi: 10.1371/journal.pgen.1007317 (PMC5897033; doi:10.1371/journal.pgen.1007317)

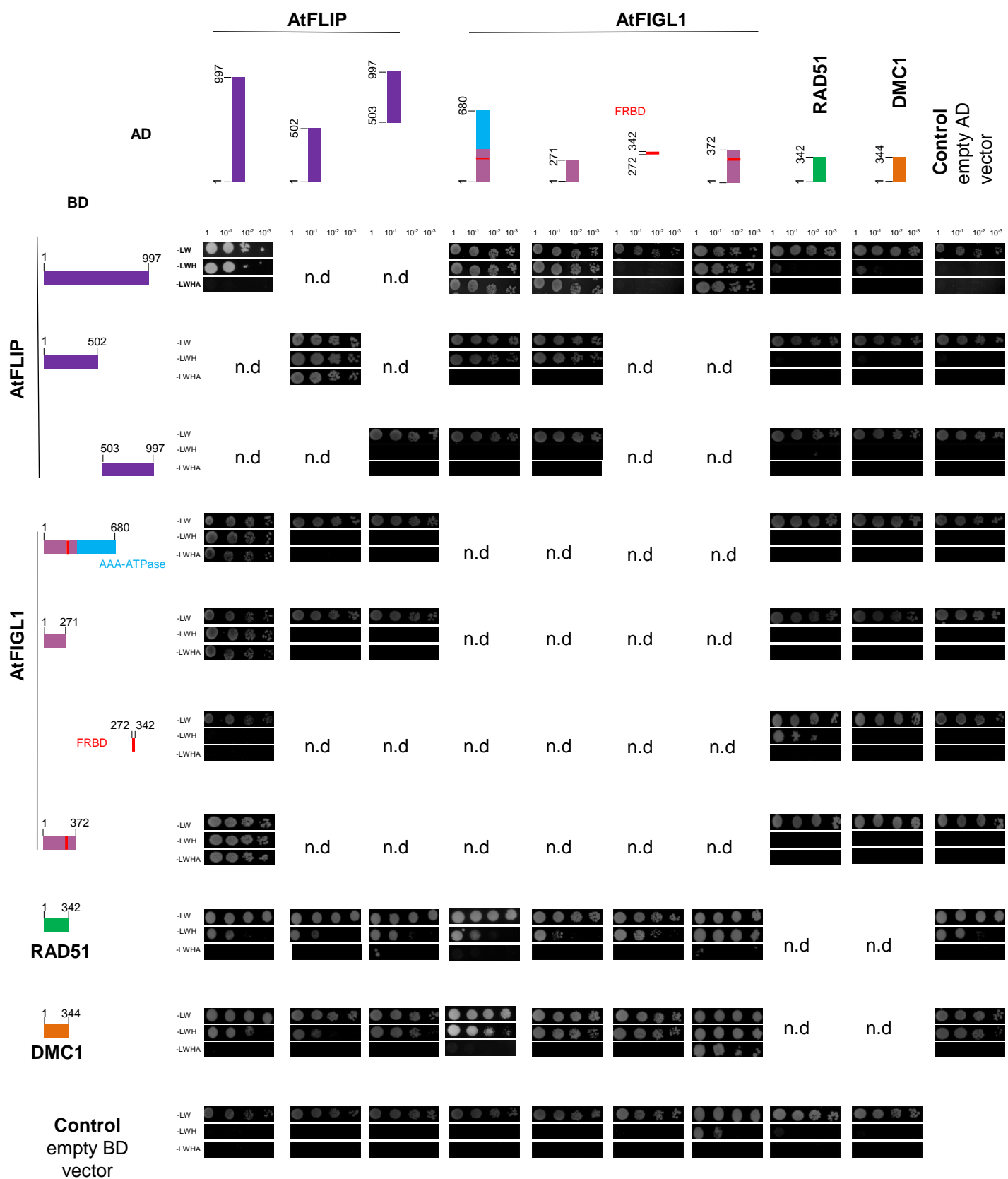

Supplement: S1 Fig — Proteins of interest were fused with Gal4 DNA binding domain (BD) and with Gal4 activation domain (AD), respectively and co-expressed in yeast cells. For each combination, serial dilutions of yeast cells were spotted on non-selective medium (-LW), moderately selective media (-LWH) and more selective media (-LWHA). Growth on LWH is interpreted as weak interaction and growth on LWHA is interpreted as strong interaction. (PDF) [file pgen.1007317.s001.pdf]

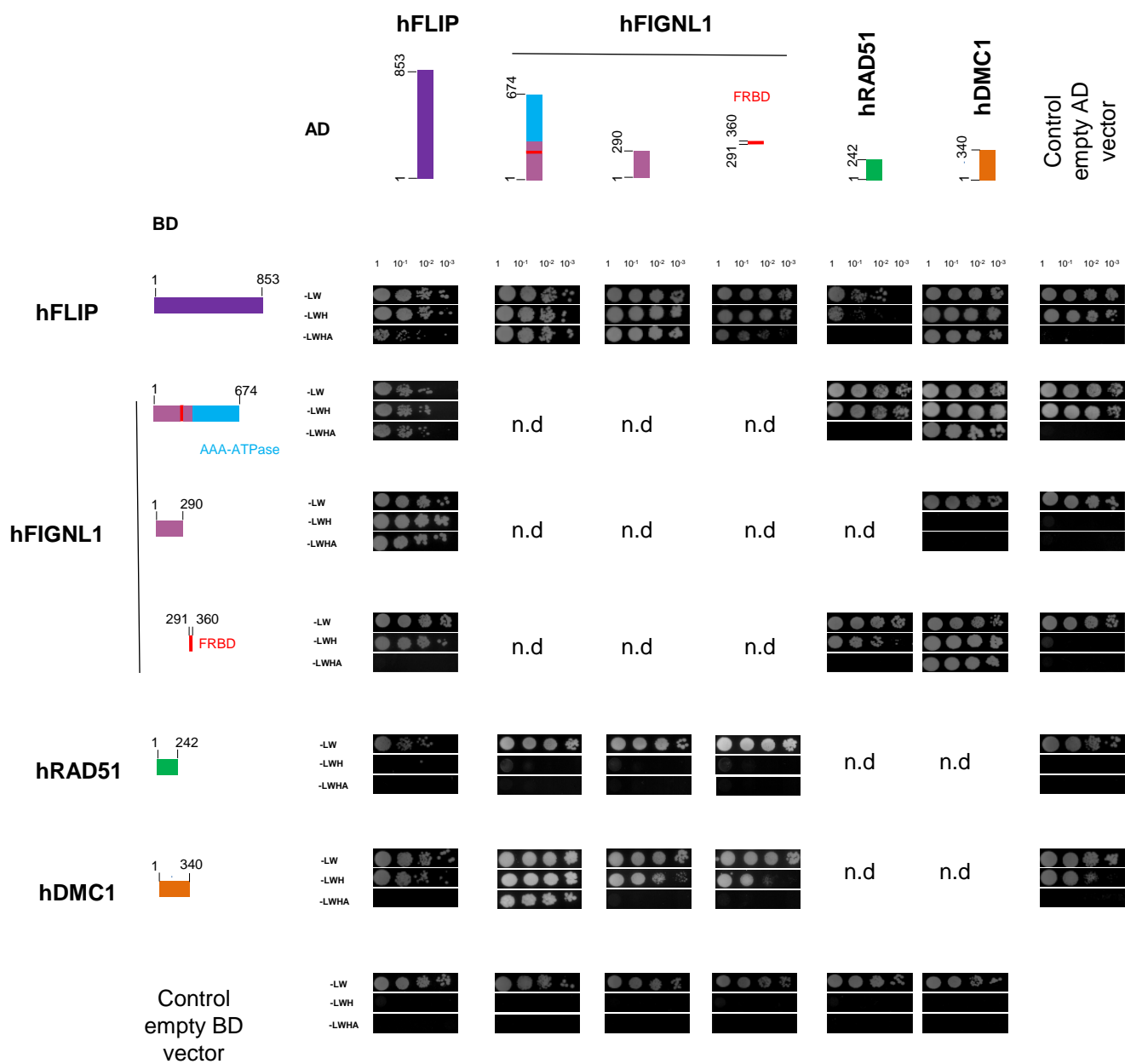

Supplement: S2 Fig — Proteins of interest were fused with Gal4 DNA binding domain (BD) and with Gal4 activation domain (AD), respectively, and expressed in yeast cells. For each combination, serial dilutions of yeast cells were spotted on non-selective medium (-LW), moderately selective media (-LWH) and more selective media (-LWHA). Growth on LWH is interpreted as weak interaction and growth on LWHA is interpreted as strong interaction. (PDF) [file pgen.1007317.s002.pdf]

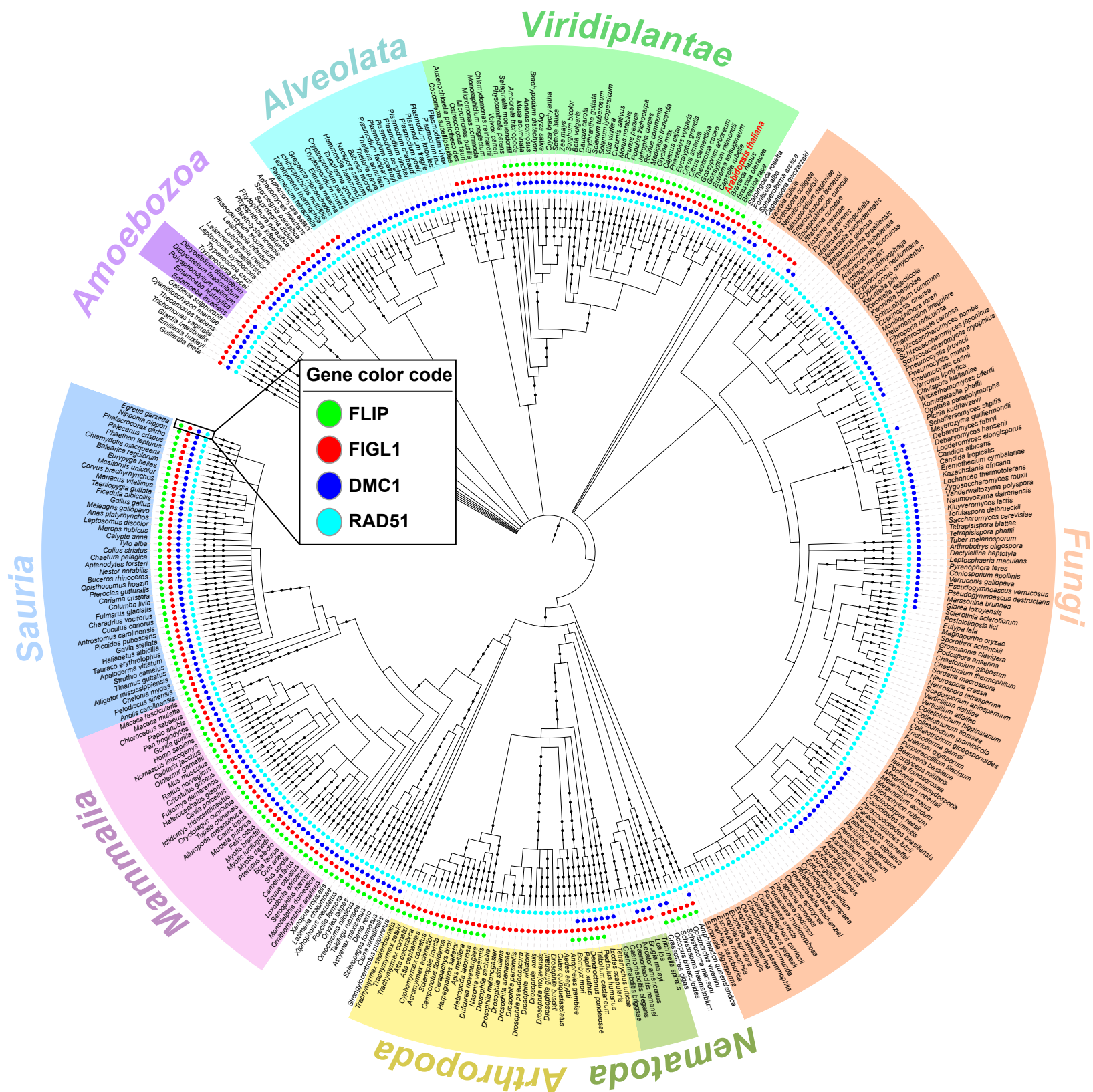

Supplement: S3 Fig — All the NCBI and JGI gene entries are listed in S2 Table and can be retrieved from the interactive tree (http://itol.embl.de/tree/132166555992271498216301). (PDF) [file pgen.1007317.s003.pdf]
